# Effects of family planning service utilization determinants on unmet need incidents with generalized structural equation modeling

**DOI:** 10.1016/j.xagr.2022.100088

**Published:** 2022-08-24

**Authors:** Ayudina Larasanti, Dumilah Ayuningtyas

**Affiliations:** Faculty of Public Health, Department of Health Policy and Administration, Universitas Indonesia, Depok, Indonesia

**Keywords:** family planning, generalized structural equation modeling, health care, Indonesian Demographic and Health Survey, medical facility, reproductive, service quality, unmet need, woman health, women's autonomy

## Abstract

**BACKGROUND:**

One of the reasons for the lack of optimal achievement in terms of contraceptive use in Indonesia is the unmet need for family planning that has not been fully addressed.

**OBJECTIVE:**

This study aimed to determine the direct, indirect, and total effect of the determinants of family planning services utilization on the incidence of unmet need spacing and limiting in Indonesia to provide a basis for appropriate and effective program recommendations.

**STUDY DESIGN:**

This study used secondary data from the 2017 Indonesian Demographic and Health Survey with a cross-sectional design with 33,635 respondents who were married women aged 14 to 49 years.

**RESULTS:**

The results of the generalized structural equation modeling showed that the incidence of unmet need in Indonesia was 11.68%, consisting of unmet need spacing (4.86%) and unmet need limiting (6.82%). The quality of family planning services had a positive indirect effect on the incidence of unmet need spacing. The quality of family planning services also had a positive direct, indirect, and total effect on the incidence of unmet need limiting. Socioeconomic and demographic status variables had a negative direct effect on the incidence of unmet need spacing, whereas the indirect and the total effect were positive. The direct and total effect of socioeconomic and demographic status variables on the incidence of unmet need limiting were positive. The women's autonomy variable had a positive direct, indirect, and total effect on the incidence of unmet need spacing. Women's autonomy had positive indirect and total effect on the incidence of unmet needs limiting. The variable of access problems did not have a significant effect on the incidence of unmet need spacing and unmet need limiting. The ideal number of children had a negative direct and total effect on the incidence of unmet need spacing and unmet need limiting in Indonesia.

**CONCLUSION:**

The variables that have been shown to have a significant effect on the incidence of unmet needs were the quality of family planning services, socioeconomic and demographic status, women's autonomy, and the ideal number of children.

## Highlights

• The magnitude and direction of direct and indirect effects can be analyzed by generalized structural equation modeling.
• Socioeconomic status has the greatest direct and total effect on unmet need.
• Socioeconomic status has the greatest indirect effect on unmet need spacing.
• Quality of family planning services has the greatest indirect effect on unmet need limiting.

**AJOG Global Reports at a Glance**

**Why was this study conducted?**

Optimum contraceptive use has not yet been achieved in Indonesia, partly because the unmet need for family planning has not been fully overcome. The health impact of the high unmet need for birth control is enormous.

**Key findings**

Socioeconomic status has the greatest direct and total effect on unmet need for family planning.

**What does this add to what is known?**

The magnitude and direction of direct and indirect effects can be analyzed by generalized structural equation modeling. Socioeconomic status has the greatest indirect effect on unmet need spacing, and the quality of family planning services has the greatest indirect effect on unmet need limiting.

The 2019 revision of the World Population Prospects issued by the Population Division of the United Nations Department of Economic and Social Affairs reported that the world population reached 7.7 billion in mid-2019.^1^ According to Worldometer (2020), Indonesia has a population of 273.5 million people, placing it as the country with the fourth largest population globally after China, India, and the United States.^2^ One of the factors that contribute to this population size is the total fertility rate (TFR). In 2019, Indonesia's TFR amounted to 2.45 children per woman, an increase from 2.38 children per woman in 2018.^3^ Optimum contraceptive use in Indonesia has not yet been achieved, partly because the unmet need has not been fully overcome.

The unmet need for family planning (FP) refers to a situation in which there is desire to avoid or delay birth, but contraceptive methods are not being used.^4^ According to the Population Reference Bureau (2019), estimates of unmet needs can help identify where the largest resources need to be invested in FP programs.^5^ The results from the Indonesia Demographic and Health Survey (IDHS) showed that there has been no significant change in the 11% incidence of unmet need for FP in Indonesia between 2012 and 2017.^6^^,^^7^ The results of the Program Performance and Accountability Survey showed that, in 2019, this percentage increased to 14.4%. Given the national target of 9.91% set by the 2015–2019 Strategic Planning of the National Population and Family Planning Board (BKKBN), these numbers are still far from reaching the specified goals.^3^

The 2017 IDHS used a new definition of unmet need that has been in use since the 2012 IDHS. As described in the 2012 IDHS report, thus far calendar calculations have not been used by all countries, and consequently unmet need must be defined differently for countries such as Indonesia that include calendar calculations. To make the definition of unmet need comparable in both types of surveys, the new definition does not take information on contraceptive failure into account for any woman when assigning unmet need status. Removing contraceptive failure from the calculation results in a small increase in the estimated level of unmet need over the level that would be obtained using the previous definition in countries such as Indonesia in which calendar data are available. This calculation change has been made since the 2012 IDHS and continued until the last 2017 IDHS.^6^^,^^7^

The health impact of the high unmet need for birth control is significant. Supposing that all unmet needs for modern FP methods were met, 52 million unwanted pregnancies could have been prevented, thereby preventing the deaths of 70,000 women from pregnancy-related causes (eliminating 18,000 deaths from unsafe abortions and 53,000 deaths caused by other complications of pregnancy and childbirth).^8^ The phenomenon of unmet need is multidimensional because it is affected by various factors, such as demographic characteristics, socioeconomics, attitudes, access, and quality of service.^9^ Unmet need can be a valuable indicator of the progress toward achieving universal access targets for reproductive health. It becomes important to research the direct effect and indirect effect determinants of the utilization of FP services in the incidence of unmet need by further analyzing IDHS 2017 data using generalized structural equation modeling (GSEM), which simultaneously models the direct and indirect effects of the interacting factors and thus provides better results in observational studies.^10^

## Materials and Methods

This study used a cross-sectional design with a quantitative research approach using secondary data from the 2017 IDHS (the most recent study conducted in Indonesia) from all 34 provinces, with permission to use data obtained by applying for a permit and downloading it on the website https://dhsprogram.com/. Ethical clearance was received from the Research Ethics Commission and Community Service, Faculty of Public Health, Universitas Indonesia (ethical review number Ket-196/UN2. F10.D11/PPM.00.02/2021). The IDHS raw data were then processed by editing, cleaning, and recoding variables needed for further analysis to obtain meaningful information related to research questions.

According to Ramlall,^11^ it is vital to ensure that the sample size is large enough to produce accurate statistical results in analyzing data using structural equation modeling (SEM). The minimum ideal sample number in SEM is a 20:1 ratio of the number of subjects to the number of model parameters.^11^ In this study, there were 12 observed variables, thus the minimum sample size that could be used for the SEM analysis was 12 × 20=240 samples. The total sample that went through the weighting process and met the inclusion and exclusion criteria of the study consisted of 33,635 women, which was the number ultimately used in this study. The sample used raw data from the 2017 IDHS that were weighted because the 2017 IDHS sampling design uses a complex sample, namely stratified sampling with ≥2 sampling methods. The weighting aimed to return the sample to population proportions because the IDHS sampling is oversampling; for example, in Eastern Indonesia the proportion was too small, thus additional samples were added. In cases of undersampling (eg, on the island of Java, where the number of samples was too large), a sample reduction was carried out so that the proportions remained comparable.

The data analysis in this study consisted of univariate and GSEM analysis, using Stata 17 (StataCorp LLC, College Station, TX) to examine the direct effect and indirect effect of independent variables (determinants of utilization of FP services consisting of variables of quality of FP services, socioeconomic and demographic status [SES], women's autonomy, problem of access to health services, and the ideal number of children) on the dependent variable (unmet need incidence) in women aged 15 to 49 years.

More specifically, the variable of quality of FP services was obtained through several indicators, namely FP officer visits, FP information from mass media, FP information from health visits, and FP information from other sources. The more FP services the respondent received, the more likely it was that she will be classified as a recipient of high-quality FP services (if the respondent stated that she had received 3 out of 4 types of FP services, which was a determining indicator of the variable of quality of FP services).

The variable of women's autonomy was obtained through 3 types of determining indicators, namely participation in decision-making, attitudes related to violence against wives, and attitudes of refusing to have sex. The problem of access to health services variable was obtained from several indicators, including problems related to getting permission for treatment, treatment costs, distance to health facilities, and not wanting to go alone. All of these indicators are contained in the 2017 IDHS.

SEM is a multivariate statistical analysis technique used to analyze measurement relationships and structural relationships of a number of variables. SEM is an extension of the previously known linear and logistic regression models, involving not only direct relationships as occurs in regression models, but also indirect relationships between variables.^12^ GSEM is a framework that combines the power of SEM and the generalized linear model, making it possible to model continuous and discrete variables together in the same latent construct under various assumptions and probability distributions.^13^

## Results

The univariate analysis showed that there were as many as 1634 (4.86%) women in Indonesia who had unmet need spacing, and as many as 2293 (6.82%) who had unmet need limiting, making the total number of women who had unmet need 3927 (11.68%). The univariate analysis also showed that most respondents received low-quality FP services (71.72%), including in the category of old age (35–49 years; 54.04%), low education (85.3%), high wealth index (57.43%), number of children still alive of 0 to 2 (65.53%), working (56.8%), low husband education (86.36%), urban dwelling (50.22%), low autonomy (69.83%), low problem of access to health care (94.86%), and an ideal number of children ≥3 (51.68%).

### Generalized structural equation modeling

**Figure 1:**
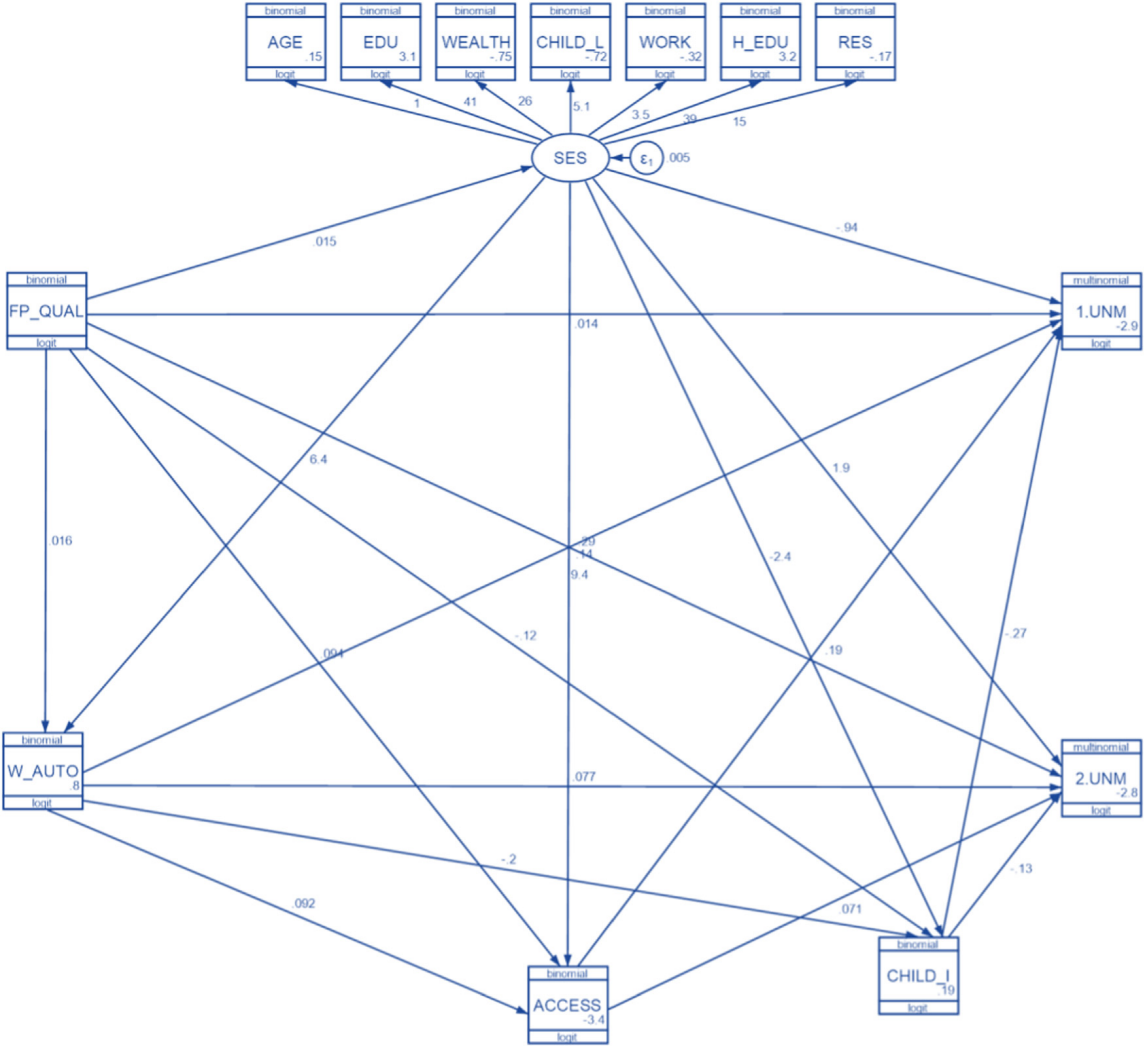
GSEM model *GSEM*, generalized structural equation modeling.

The model formed belongs to the category of overidentified (degree of freedom value >0). The confirmatory factor analysis results showed that all indicators had a significant contribution to SES variables (*P*<.05). The next step was to estimate the overall GSEM model, presented in the Figure 1, and variable descriptions are presented in Table 1, which integrates the following.

Larasanti. Effects of family planning service utilization determinants on unmet need incidents with generalized structural equation modeling. Am J Obstet Gynecol Glob Rep 2022.

**Table 1:** Description of variables used in generalized structural equation modeling

Variable	Description	Value
SES	Socioeconomic status	—
AGE	Age	Young (15–34), Old (35–49)
EDU	Education	High, Low
WEALTH	Family wealth index	High, Low
CHILD_L	Number of children still alive	0–2 children, ≥3 children
WORK	Respondents’ working status	Working, Not working
H_EDU	Husband's education	High, Low
RES	Residence	Urban, Rural
FP_QUAL	Quality of FP service	High, Low
W_AUTO	Women's autonomy	High, Low
ACCESS	Problem of access to health care	Low, High
CHILD_I	The ideal number of children	≥3 children, ≤2 children
1.UNM	Unmet need spacing	No, Yes
2.UNM	Unmet need limiting	No, Yes

The base outcome was no unmet need.

*FP*, family planning.

*Larasanti. Effects of family planning service utilization determinants on unmet need incidents with generalized structural equation modeling. Am J Obstet Gynecol Glob Rep 2022.*

On the basis of Table 2 it can be determined that in the estimation of measurement, the latent variable of SES, all indicators have a *P* value <.05, which means all indicators had a significant contribution to the SES variable. The largest contribution was from the maternal education indicator, with a loading factor of 40.84. In contrast, the smallest contribution indicator is the age indicator, with a loading factor of 1. In structural models, the incidence of unmet need spacing in Indonesia is affected directly and significantly by women's autonomy and ideal number of children. Low women's autonomy will increase the incidence of unmet need spacing. Women with low autonomy had unmet need spacing 0.14 units higher than women with high autonomy (B, 0.14; 95% confidence interval [CI], 0.02–0.25; *P*=.02). An ideal number of children of ≤2 decreased the incidence of unmet need spacing. Women with an ideal number of children of ≤2 had unmet need spacing 0.27 units lower than women with an ideal number of children of ≥3 (B, −0.27; 95% CI, −0.38 to −0.17; *P*<.001).

The incidence of unmet need limiting in Indonesia is directly and significantly affected by the quality of FP services, SES, and the ideal number of children. Low quality of FP service will increase the incidence of unmet need limiting. Women who received low-quality FP services had unmet need limiting 0.29 units higher than women who received high-quality FP service (B, 0.29; 95% CI, 0.19–0.39; *P*<.001). Low SES increased the incidence of unmet need limiting. Women with low SES had unmet need limiting 1.86 units higher than women with high SES (B, 1.86; 95% CI, 0.81–2.90; *P*=.001). An ideal number of children of ≤2 decreased the incidence of unmet need limiting. Women with an ideal number of children ≤2 had unmet need limiting 0.13 units lower than women with an ideal number of children ≥3 (B, −0.13; 95% CI, −0.22 to −0.04; *P*=.003).

Table 3 shows that variables that had significant indirect effects were FP service quality (B, 0.098; 95% CI, 0.04–0.16; *P*=.002), SES (B, 3.74; 95% CI, 0.98–6.50; *P*=.008) and women's autonomy variables (B, 0.07; 95% CI, 0.34–0.11; *P*<.001). Variables that had a significant total effect on the incidence of unmet need spacing were the SES variables, women's autonomy, and the ideal number of children. The variable with the greatest total effect with a significant positive orientation to the occurrence of unmet need spacing in Indonesia was the SES variable (B, 2.80; 95% CI, 0.24–5.36; *P*=.032).

Related to unmet need limiting in Indonesia, variables that had a significant indirect effect were FP service quality (B, 0.08; 95% CI, 0.03–0.13; *P*=.002) and women's autonomy (B, 0.03; 95% CI, 0.01–0.06; *P*=.016). Variables that significantly affected the incidence of unmet need limiting were variables in the quality of birth control services, SES, women's autonomy, and the ideal number of children. The variable that had the greatest total effect with a significant positive orientation to the occurrence of unmet need limiting in Indonesia was the SES variable (B, 3.53; 95% CI, 1.14–5.92; *P*=.004).

**Table 2:** Generalized structural equation modeling analysis of the effect of utilization of family planning services on unmet need incidence in Indonesia

Dependent variables	Independent variables	B	95% CI	*P* value
Structural				
No unmet need (base outcome)				
Unmet need spacing				
Unmet need spacing	← Quality of FP service	0.01	−0.10 to 0.13	.806
	← SES	−0.94	−1.92 to 0.04	.061
	← Women's autonomy	0.14	0.02–0.25	.020^a^
	← Problem of access	0.19	−0.03 to 0.40	.090
	← Ideal number of children	−0.27	−0.38 to −0.17	<.001^a^
Unmet need limiting				
Unmet need limiting	← Quality of FP service	0.29	0.19–0.39	<.001^a^
	← SES	1.86	0.81–2.90	.001^a^
	← Women's autonomy	0.08	−0.02 to 0.18	.127
	← Problem of access	0.07	−0.11 to 0.26	.448
	← Ideal number of children	−0.13	−0.22 to −0.04	.003^a^
SES	← Quality of FP service	0.02	0.009–0.02	<.001^a^
Women's autonomy	← Quality of FP service	0.02	−0.04 to 0.07	.545
	← SES	6.40	3.67–9.13	<.001^a^
Problem of access	← Quality of FP service	0.09	−0.02 to 0.21	.113
	← SES	9.39	5.30–13.47	<.001^a^
	← Women's autonomy	0.09	−0.03 to 0.21	.135
The ideal number of children	← Quality of FP service	−0.12	−0.17 to −0.07	<.001^a^
	← SES	−2.36	−3.40 to −1.33	<.001^a^
	← Women's autonomy	−0.20	−0.25 to 0.15	<.001^a^
Measurement				
Age	← SES	1		
Education	←	40.84	23.92–57.76	<.001^a^
Wealth index	←	25.90	14.65–37.15	<.001^a^
Number of children still alive	←	5.08	3.05–7.12	<.001^a^
Working status	←	3.46	1.91–5.02	<.001^a^
Husband's education	←	39.08	22.62–55.55	<.001^a^
Residence	←	14.90	8.51–21.29	<.001^a^
Type				
N observation	= 33,635			
Df	= 38			
Log likelihood	= −199,163.7			
AIC	= 398,403.4			
BIC	= 398,723.5			

*AIC*, Akaike information criterion; *BIC*, Bayesian information criterion; *CI*, confidence interval; *Df*, degrees of freedom; *FP*, family planning; *SES*, socioeconomic and demographic status.

*Larasanti. Effects of family planning service utilization determinants on unmet need incidents with generalized structural equation modeling. Am J Obstet Gynecol Glob Rep 2022.*

a Significant at α level of 5%.

**Table 3:** Indirect and total influence of family planning service utilization on unmet need incidence in Indonesia

Variable	Indirect influence (95% CI)	*P* value	Total influence (95% CI)	*P* value
No unmet need (base outcome)				
Unmet need spacing				
Quality of FP service	0.098 (0.04–0.16)a	.002a	0.11 (−0.01 to 0.24)	.082
SES	3.74 (0.98–6.50)a	.008a	2.80 (0.24–5.36)a	.032a
Women's autonomy	0.07 (0.34–0.11)a	<.001a	0.21 (0.09–0.33)a	.001a
Problem of access	—	—	0.19 (−0.03 to 0.4)	.09
The ideal number of children	—	—	−0.27 (−0.38 to −0.17)a	<.001a
Unmet need limiting				
Quality of FP service	0.08 (0.03–0.13)a	.002a	0.37 (0.25–0.48)a	<.001a
SES	1.68 (−0.45 to 3.80)	.122	3.53 (1.14–5.92)a	.004a
Women's autonomy	0.03 (0.01–0.06)a	.016a	0.10 (0.01–0.21)a	.035a
Problem of access	—	—	0.07 (−0.11 to 0.26)	.448
The ideal number of children	—	—	−0.13 (−0.22 to −0.04)a	.003a

*CI*, confidence interval; *FP*, family planning; *SES*, socioeconomic and demographic status.

^a^Significant at α level of 5%.

## Discussion

The results showed that 11.68% of women of childbearing age (15–49 years) in Indonesia experienced an unmet need incident, consisting of unmet need spacing (4.86%) and unmet need limiting (6.82%). These results are not much different from those of the IDHS 2012 and 2017 reports.^6^^,^^7^ Various policies related to the decrease in unmet need and the strategy of increasing contraceptive user coverage have been carried out by the government, both in the 5 years before IDHS 2012 and in the 5 years before IDHS 2017. However, various obstacles still exist and cannot be eliminated from both periods, one of which is the commitment of local governments that vary in their recognition of the importance of the FP program. Problems related to the low commitment of regional heads in supporting the FP program are contained in the Program Final Evaluation Report issued by Bappenas^14^ in 2010 and are still found in the BKKBN Performance Reports for 2017, 2018, 2019, and 2020.^14^^,^^15^ Discussions related to direct, indirect, and total effects of each determinant variable on the utilization of FP services in unmet need incidents are as follows.

### Quality of family planning service

As explained in the methods section, variable of quality of FP services in this study was obtained through several indicators, namely FP officer visits, FP information from the mass media, FP information from health visits, and FP information from other sources. The more FP services the respondent received, the more likely it was that the respondent will be classified as a recipient of high-quality FP services. The quality of FP services had a statistically significant effect with a positive direction to unmet need incidents in Indonesia. Low quality of FP services increased the incidence of unmet need limiting and unmet need spacing in Indonesia. This result is similar to those of Masitha Aulia's study^16^ (2020), in which women who received low-quality FP services had a 3.3 times greater risk of having an unmet need compared with those who received high-quality FP services. Taufiqoh's research^17^ (2020) also found that women who discussed FP with FP field officers had significantly less tendency to have unmet needs.

There are various obstacles faced by regions in Indonesia in improving the quality of FP services, one of them being the number of women of childbearing age who do not get visits from FP officers. The high percentage of women who do not get FP officer visits is likely due to the limited number of FP officers. The current ratio of FP officers to villages is 1:5, which means that 1 officer is responsible for 5 villages, whereas the ideal ratio would be 1:2. The overly large task area poses obstacles to FP officers in reaching women of childbearing age.^18^

Efforts in reducing the incidence of unmet needs and increasing the use of contraception would be facilitated if the provision of FP information through all types of mass media were improved. Level of media exposure is known to have a positive relationship with the use of modern contraceptives.^19^ The choice of FP methods and sources of FP information can help reduce unmet needs and ultimately reduce maternal and infant mortality. In addition, broader counseling and FP education efforts can eliminate the unwarranted fear of side effects of contraceptive practices, and this is one way to increase the use of contraception and reduce unmet need.^20^ In addition to expanding the scope of information, the quality and type of basic FP information must also be considered, especially explanations or information related to side effects and handling and other FP method choices.

### Socioeconomic and demographic status

Latent variables of SES consisting of indicators of age, education, wealth index, number of children still alive, working status, husband's education, and residence had a significant effect with a positive direction on incidents in Indonesia, both unmet need spacing and unmet need limiting. These results are similar to those of a previous study that found that the level of unmet need increased with age.^21^ Higher-educated women were less likely to have unmet contraceptive needs. Women who had ≥5 surviving children were more likely to have unmet contraceptive needs.^22^ An increase in household wealth levels was negatively related to unmet need.^19^ Working women were less likely to have unmet needs.^23^ There was a positive relationship between couples’ education and the use of contraceptive methods.^24^ The need for FP is usually higher in rural areas for both young married and unmarried women.^25^ The main concern should be given to women with old age, low education, a low wealth index, a number of children still alive of ≥3, and low husband education, and those not working and living in rural areas. Ease in accessing FP services, in terms of both affordability and costs, can reduce the incidence of unmet need, especially in women of low SES.

### Women's autonomy

Women's autonomy had a significant effect with a positive orientation to the occurrence of unmet needs in Indonesia, in terms of both unmet need spacing and unmet need limiting. The results of this study are similar to those of Solanke's^23^ study, which showed that women's autonomy was significantly related to unmet need. The probability of unmet need was reduced to 0.601 in women with moderate autonomy and subsequently dropped to 0.467 among women with high autonomy.^23^) Amraeni^26^ also showed an increase in unmet needs limiting among women with low autonomy. The higher the women's reproductive autonomy, the lower the chances of having unmet needs limiting in urban and rural areas.^26^

Gender equality can affect contraceptive use in many ways. Women can have the freedom to choose their sexual activities and access to FP services. Reproductive health and women's empowerment are interrelated, thus trends in contraceptive use can also show progress toward other global targets of gender equality.^27^ The importance of women's autonomy in the use of FP services and the decline of unmet need shows that the involvement of husbands/couples in education related to FP and reproductive health is important. Husbands/partners who realize the importance of women's autonomy and gender equality will help women be freer in determining their reproductive rights, including whether or not to decide to have a child, and the types of contraceptive methods that women want to use. High women's autonomy will decrease the incidence of unmet need spacing and unmet need limiting.

### Problem of access to health services

In this study, the indicators used in determining the variable of problems of access to health services were from the 2017 IDHS/DHS VII questionnaire. Univariate analysis showed that most respondents had low problems with access to health services (94.86%), indicating that in general, women of childbearing age in Indonesia do not experience health access problems. This quite dominant percentage is a possible cause that statistically access problem variables do not have a significant relationship with the incidence of unmet need spacing and unmet need limiting in Indonesia.

Although the results of this study showed that the problem of access to health services does not significantly affect the incidence of unmet need spacing or unmet need limiting in Indonesia, this is not a reason to ignore the access problem factor. Experts agree that access is an important element in health care and contraception, and is specifically related to decreasing the incidence of unmet needs. According to Pratiwi and Basuki,^28^ access affects women's health-seeking behavior in FP services, especially with regard to nearby healthcare facilities, practice midwives, and village midwives. Paragraph 78 of Law No. 36 of 2009 on Health states that the government is responsible for and ensures the availability of personnel, service facilities, tools, and drugs in providing FP services that are safe, high-quality, and affordable to the community.^29^

Postpartum FP services are an effective step in efforts to reduce the incidence of unmet need both in women in areas that have difficulty accessing health services because of distance and in women who live and work in big cities that deprive them of time to visit health facilities every month or once every 3 months to access FP services. Data from the Directorate General of Public Health, Ministry of Health (as of March 3, 2020) contained in the Indonesian Health Profile 2019 reported that the coverage and proportion of postpartum FP in Indonesia amounted to 34.3%.^30^ With regard to efforts to reduce unmet need, it is necessary to increase the coverage of postpartum FP by minimizing various problems found in the field related to postpartum FP services.

### Ideal number of children

According to the analysis results, an ideal number of children of ≤2 decreased the incidence of unmet need spacing and unmet need limiting. This was similar to the finding of Adebowale and Palamuleni^31^ that women who want more children and are of reproductive age only want contraception to regulate birth spacing. Conversely, some married women who cited 1 to 2 children as the ideal number must have had a desire to limit births both at this time and in the past but did not have access to the modern contraceptives of their choice.^31^

## Conclusion

The incidence of unmet need in women aged 15 to 49 years in Indonesia amounted to 11.68%, consisting of unmet need spacing (4.86%) and unmet need limiting (6.82%). These results were not significantly different from those of IDHS 2012 in terms of the overall numbers of unmet need incidents, unmet need spacing incidents, and unmet need limiting incidents. There were significant determinants of utilization of FP services in the occurrence of unmet need, including the quality of FP services, SES, women's autonomy, and the ideal number of children. However, access problems did not significantly affect the occurrence of unmet needs in Indonesia.

### Recommendations

There is urgent need to improve the quality of FP services, paying more attention to women of low SES, low autonomy, high access problems, and an ideal number of children of ≥3. Postpartum FP is the most effective way to reduce the incidence of unmet need. The use of mass media in the provision of FP information, especially related to the side effects of specific methods, can be improved. Moreover, it can be adapted to popular Indonesian television shows and mass media platforms that are currently widely used by the public. There could be great benefit in using various existing social media platforms, such as YouTube, Instagram, Facebook, Twitter, and TikTok, for promoting health education in general, including about the importance of FP. Continuously exposing people to information about FP is likely to decrease the incidence of unmet need for FP. It is also necessary to increase the active role of health workers in providing integrated FP counseling in maternal and child health services.
